# Hybrid Close-Loop Systems Versus Predictive Low-Glucose Suspend and Sensor-Augmented Pump Therapy in Patients With Type 1 Diabetes: A Single-Center Cohort Study

**DOI:** 10.3389/fendo.2022.816599

**Published:** 2022-04-14

**Authors:** Maria Elena Lunati, Paola Silvia Morpurgo, Antonio Rossi, Alessandra Gandolfi, Irene Cogliati, Andrea Mario Bolla, Laura Plebani, Luciana Vallone, Laura Montefusco, Ida Pastore, Vincenzo Cimino, Sabrina Argenti, Graziella Volpi, Gian Vincenzo Zuccotti, Paolo Fiorina

**Affiliations:** ^1^Endocrinology Division, Azienda Socio Sanitaria Territoriale (ASST) Fatebenefratelli Sacco, Milan, Italy; ^2^International Center for T1D, Centro di Ricerca Pediatrica Romeo ed Enrica Invernizzi, Department of Biomedical and Clinical Science “L. Sacco”, University of Milan, Milan, Italy; ^3^Centro di Ricerca Pediatrica Romeo ed Enrica Invernizzi, Dipartimento di Scienze Biomediche e Cliniche “L. Sacco”, Università di Milano, Milan, Italy; ^4^Dipartimento di Pediatria, Ospedale dei Bambini Buzzi, Milan, Italy; ^5^Nephrology Division, Boston Children’s Hospital, Harvard Medical School, Boston, MA, United States

**Keywords:** T1D, HCL, insulin pump, SAP, PLGS, time in range

## Abstract

**Introduction:**

Predictive low-glucose suspend (PLGS) and hybrid closed-loop (HCL) systems may improve glucose control and quality of life in type 1 diabetic individuals. This is a cross-sectional, single-center study to compare the effect on metabolic control and glucose variability of PLGS and HCL systems as compared to standard sensor-augmented pump (SAP) therapy.

**Methods:**

We retrospectively analyzed 136 adults (men/women 69/67, mean age 47.3 ± 13.9 years) with T1D on insulin pump therapy, divided accordingly to type of insulin pump system (*group 1*: SAP, 24 subjects; *group 2*: PLGS, 49 subjects; *group 3*: HCL, 63 subjects). The groups were matched for age, gender, years of disease, years of CSII use, and CGM wear time.

**Results:**

The analysis of CGM metrics, in the three groups, showed a statistically significant different percentage of time within the target range, defined as 70–180 mg/dl, with a higher percentage in group 3 and significantly less time spent in the hypoglycemic range in groups 2 and 3. The three groups were statistically different also for the glucose management indicator and coefficient of variation percentage, which were progressively lower moving from group 1 to group 3. In the HCL group, 52.4% of subjects reached a percentage of time passed in the euglycemic range above 70%, as compared to 32.7% in those with PLGS and 20.2% in those with SAP. A positive correlation between the higher percentage of TIR and the use of auto-mode was evident in the HCL group. Finally, the three groups did not show any statistical differences regarding the quality-of-life questionnaire, but there was a significant negative correlation between CV and perceived CSII-use convenience (r = -0.207, p = 0.043).

**Conclusion:**

HCL systems were more effective in improving glucose control and in reducing the risk of hypoglycemia in patients with type 1 diabetes, thereby mitigating risk for acute and chronic complications and positively affecting diabetes technologies’ acceptance.

## Introduction

Insulin therapy in type 1 diabetes (T1D) is a burden in diabetes management. Patients have to face multiple challenges due to the complexities of insulin therapy and the variability in glucose levels from multiple factors, like meals, exercise, illness, and antecedent hypoglycemia. The last three decades showed the emergence of innovative diabetes technologies aimed at improving outcomes and easing the burden of diabetes management (1). Advantages in glucose monitoring and in insulin delivery allow better glycemic control, lower glycemic variability, and fewer hypoglycemic events (2). The development of sensor-augmented pump (SAP) therapy, which is the combination of continuous subcutaneous insulin infusion (CSII) and continuous glucose monitoring (CGM), has permitted reductions in DKA and severe hypoglycemia (3, 4). More recently, control algorithms were incorporated in SAP. These features allow the discontinuation of insulin delivery when hypoglycemia is predicted by the algorithm (PLGS—*predictive low-glucose insulin suspend*). Pumps using the algorithm were introduced in Europe and Australia in 2015 with the MiniMed 640G pump (Medtronic Diabetes), followed by a Tandem t:slim X2 insulin pump with Basal-IQ PLGS Technology. In RCTs, it has been demonstrated that the utilization of PLGS system technology reduces exposure to hypoglycemia (5, 6). In early 2017, the first hybrid close-loop (HCL) system (MiniMed 670G pump, Medtronic) was introduced in the USA, which utilizes a PID (proportional–integral–derivative) algorithm with insulin feedback (7). In *auto-mode*, this system can provide automated glucose-responsive insulin delivery and improve the maintenance of glucose levels within a healthy range (8). Otherwise, the Control-IQ technology in the t:slim X2 pump uses a model predictive control (MPC) algorithm that predicts future glucose levels based on CGM data and automatically adjusts insulin doses, aiming at keeping blood glucose levels in the target range (9, 10). Finally, the MiniMed 780G (Medtronic) is a new advanced HCL (AHCL) system that incorporates automated correction bolus doses, using the PID algorithm and fuzzy logic control (11). The aim of this study was to evaluate the effectiveness of different categories of insulin pump in maintaining improved metabolic control in T1D subjects. Moreover, we analyzed how new diabetes technology affects quality of life (QOL) and the perceived benefits by the users, in real-life settings.

## Methods

This study was a retrospective and cross-over trial, conducted at Unit of Diabetology and Endocrinology in Fatebenefratelli-Sacco Hospital, Milan, between December 2020 and June 2021. The main inclusion criteria were adult patients with type 1 diabetes aged over 18 years, who used SAP therapy for at least 6 months. Patients were divided into three groups (**Table 1**): group 1 (“SAP group”): CSII and CGM without features; group 2 (“PLGS group”): pumps with features that suspend insulin delivery before low and/or suspend, at low; and group 3 (“HCL group”): HCL and advanced HCL (AHCL) system. Key exclusion criteria were decompensated diabetes, defined as HbA1c >11% or one or more episodes of ketoacidosis requiring admission to hospital in the past 6 months, pregnancy, non-continuous use of CGM, defined as sensor wear time <60%, non-continuous use of the pump, concomitant disease that affects metabolic control or interpretation of HbA1c levels, and use of antidiabetic drugs other than insulin. Moreover, we excluded patients who did not regularly use carbohydrate counting and an insulin bolus calculator. Written informed consent was obtained from each participant, and the study was approved by the Local Ethics Committee. All participants regularly used carbohydrate counting and were individually trained regarding the features of CSII. All patients had at least a visit every 4 months. We collected data available at the last clinic visit, within the study period, including medical history, blood samples, and 14-day AGP (ambulatory glucose profile). We collected data regarding medical history, micro-macrovascular complications, and last blood analysis. Hemoglobin A1c level was measured with a Diabetes Control and Complications Trial standardized analyzer. Data regarding AGP, in particular percentage time spent in hypoglycemic (<54 mg/l and 54–69 mg/dl), euglycemic (70–180 mg/dl), and hyperglycemic (181–250 mg/dl, >250 mg/dl) ranges; CGM-measured mean glucose concentration; estimated HbA1c (eHbA1c); standard deviation (SD) and coefficient of variation (CV) of CGM-measured glucose concentrations; and percentage of sensor use and insulin requirement were collected. To assess quality of life (QOL) regarding treatment with different types of CSII, each patient completed a questionnaire for people with T1D (12), which is divided into three major areas: “Convenience” (CSII-QOL-C), “Social restrictions” (CSII-QOL-SR), and “Psychological problem” (CSII-QOL-PB). The data are expressed as mean ± SD for continuous variables, or n (%) for dichotomic variables. Differences between groups were analyzed using ANOVA or the unpaired t-test. A *post-hoc* analysis, with Bonferroni test, was applied for every ANOVA test. AGP profiles were obtained from the report of *CareLink System* (Medtronic), *Diasend*, *Clarity* (Dexcom), and *DMS Eversense* (Senseonics). All p values were two-sided. p < 0.05 was considered significant. Analyses were conducted with IBM SPSS Statistic, version 24.0 (SPSS Inc., Chicago, IL).

**Table 1:** Different types of insulin pumps used in our study.

	n (%)
**GROUP 1 (n=24)**	
Omnipod® (*Insulet Corporation*)	6 (25)
Insight (*Accu-Check*®)	7 (29.2)
Combo (*Accu-Check*®)	3 (12.5)
Solo (*Accu-Check*®)	1 (4.2)
DANA RS (*B.C. Trade*)	1 (4.2)
Equil (*B.C. Trade*)	1 (4.2)
YpsoPump (*Ypsomed*)	1 (4.2)
T:slim X2 (*Tandem Diabetes Care*)	1 (4.2)
MiniMed™ 640 g (*Medtronic*) without *Medtronic* CGM	3 (12.5)
**GROUP 2 (n=49)**	
T:slim X2 (Tandem Diabetes Care) with Basal-IQ	17 (34.7)
MiniMed™ 640 g (Medtronic)	19 (38.8)
MiniMed™ 670 g (Medtronic) without Auto-Mode	10 (20.4)
MiniMed™ 780 g (Medtronic) without SmartGuard	3 (6.1)
**GROUP 3 (n=63)**	
T:slim X2 (Tandem Diabetes Care) with Control-IQ	6 (9.5)
MiniMed™ 670 g (Medtronic)	42 (66.7)
MiniMed™ 780 g (Medtronic)	15 (23.8)

## Results

**Figure 1:**
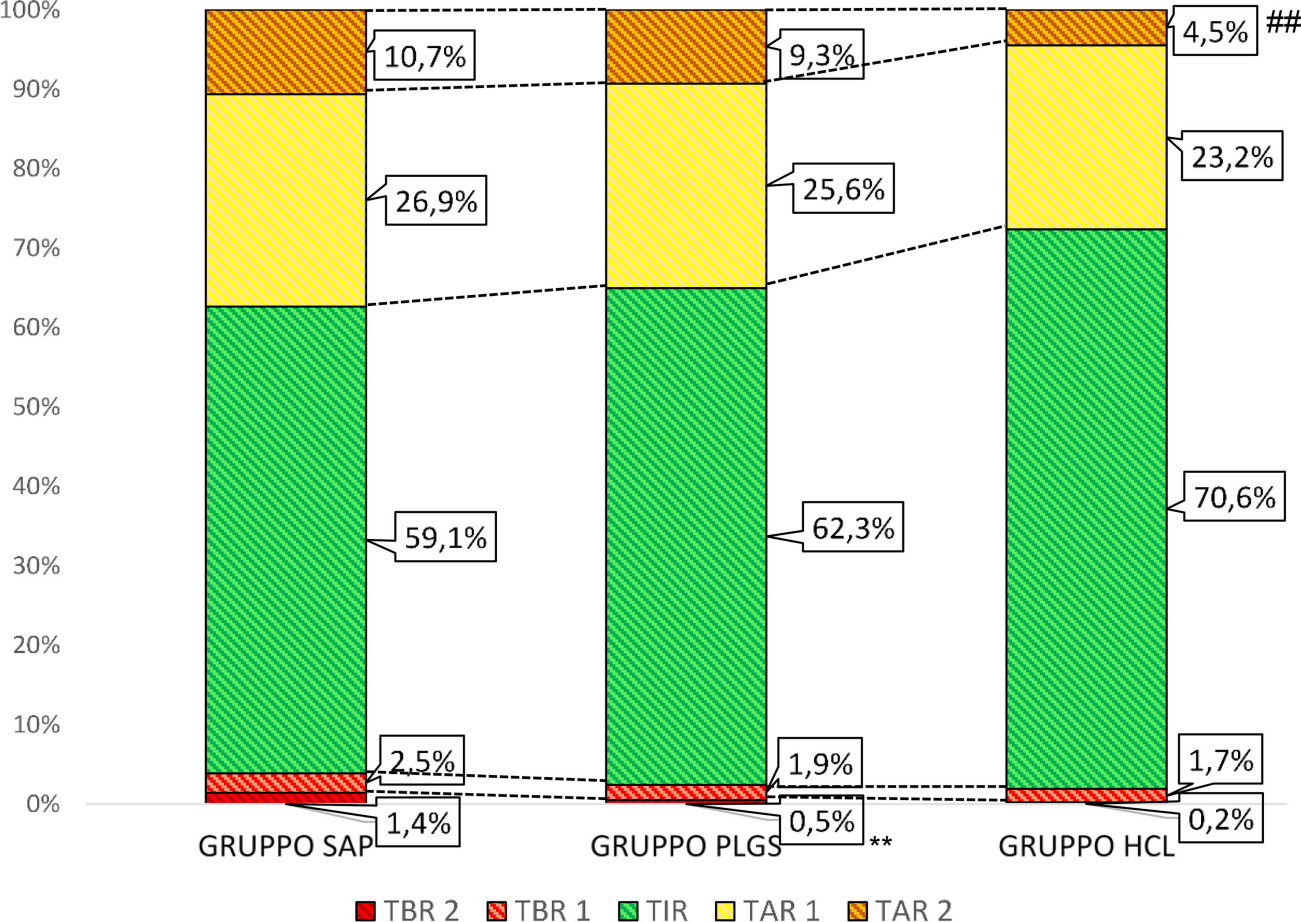
Percentage of time passed in different glycaemia ranges among the three groups of CSII. TAR 1, time above range (181–250 mg/dl); TAR 2, time above (>250 mg/dl), TBR 1, time below range (69–54 mg/dl); TBR 2, time below range (<54 mg/dl); TIR, time in range (70–180 md/dl). ** p values <0.01, HCL group versus PLGS group ## p values <0.01, PLGS group versus SAP group.

**Figure 2:**
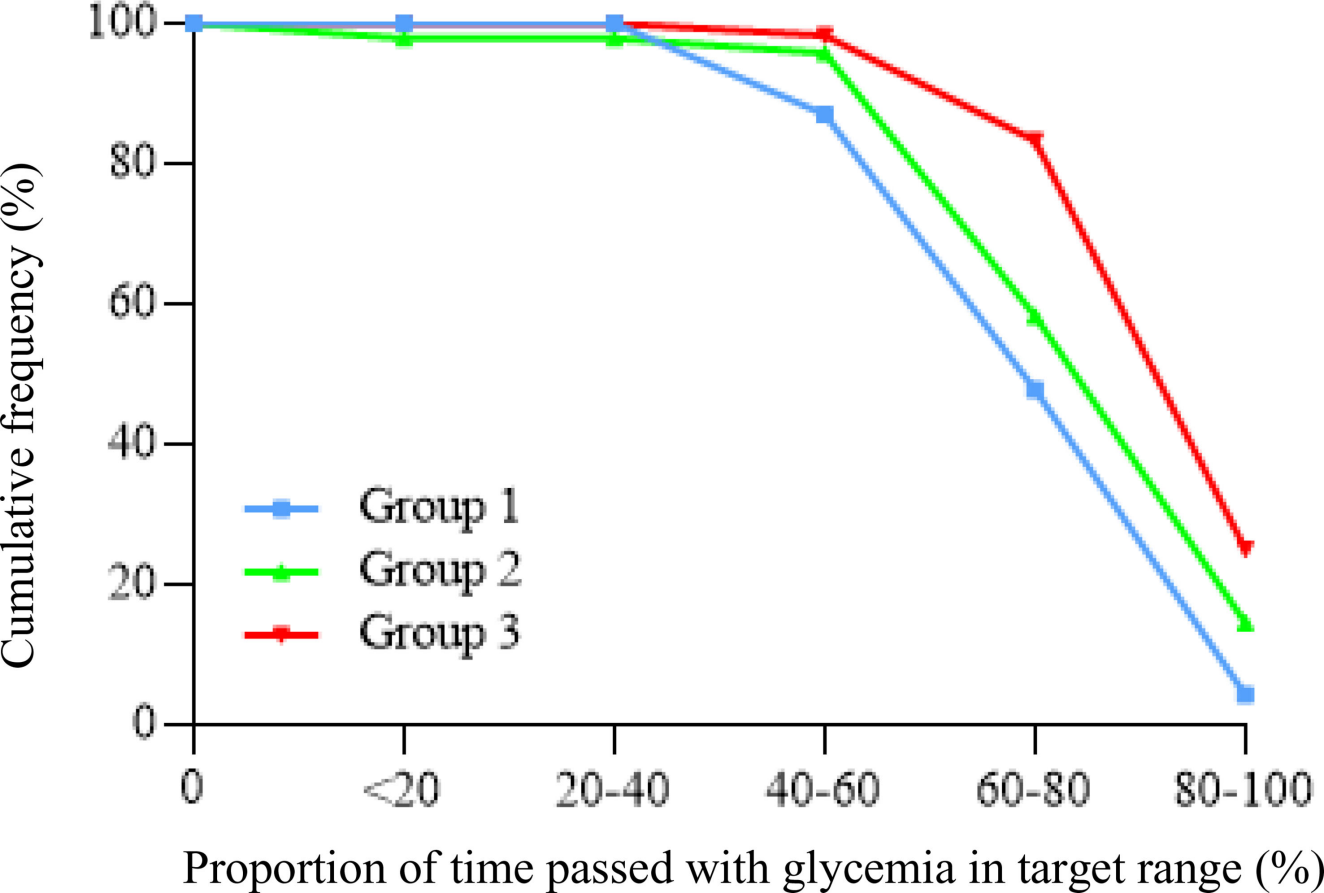
Cumulative frequency of patients reaching target time in range values (>70%) among the three groups of CSII.

The study population consisted of 136 T1D patients, men/women 69/67, the mean age was 47.3 ± 13.9 years, and the duration of diabetes was 25.6 ± 12.6 years. All subjects were divided into three groups, accordingly to characteristics of the insulin pump system used (**Table 1**). Demographic, biochemical, and anthropometric characteristics of groups as well as percentage of microvascular and macrovascular complications were similar among groups. Groups were matched for age, sex, BMI, duration of diabetes, years of CSII use, and frequency in the use of the glucose sensor (**Table 2**). All subjects had undergone SAP therapy for at least 6 months; the percentage of patients that switched from MDI to CSII in the last 12 months was 20.8% (5/24) in group 1, 28.6% (14/49) in group 2, and 38.1% (24/63) in group 3. Plasmatic HbA1c value was not statistically different among groups, even if it was lower in HCL-treated subjects. Also, the daily bolus insulin dose was slightly higher in group 1 (**Table 2**). The analysis of APG among the three groups (**Table 3**) showed a statistically significant reduction in mean glucose concentration and eHbA1c; consensually, also CV and SD progressively decreased from group 1 to group 3. The analysis of time spent in different glycemic ranges is well described in **Figure 1**. The three groups showed a progressive increase in the percentage of TIR, moving from group 1 to group 3 (**Figure 1**). TBR2, which indicates glycemia values <54 mg/dl, significantly reduced from group 1 to group 2 and from group 1 to group 3, without any statically significant difference between group 2 and group 3 (**Figure 1**). Conversely, only group 3 showed a significant reduction in glycemic values above 250 mg/dl (TAR2), compared to group 1 and group 2 (**Figure 1**). A total of 30/63 subjects (52.4%) in group 3 achieved >70% of time spent in the target range, compared to 16/49 (32.7%) in group 2 and 5/24 (20.2%) in group 1 (p = 0.003, **Figure 2**). Among patients in group 3, there was a positive correlation between time spent in auto-mode and higher percentage of TIR (r = 0.356, p = 0.009). There was no difference in the total CSII-QOL score between participants among the three groups of treatment (**Table 3**). However, we found significant and negative correlations between CV and CSII-QOL-C domain score (r = -0.207, p = 0.043).

**Table 2:** Patients characteristics.

	Group 1 (n=24)	Group 2 (n=49)	Group 3 (n=63)	P
Age (yrs)	47.1±12.1	48.4±15.9	48.6±13.1	0.91
Male (n, %)	12, 50	19, 38.7	36, 42.9	0.15
Duration of DM (yrs)	25.04±9.5	26.3±13.1	25.3±13.1	0.88
CSII use (yrs)	4.1±2.3	5.5±4.5	3.7±3.9	0.06
BMI (Kg/m^2^)	25.4±3.7	25.5±4.2	26.7±9.0	0.59
HbA1c (%)	7.6±1.4	7.3±0.8	7.2±0.7	0.24
U-Albuminuria (mg/L)	33.8±88.5	8.1±9.3	9.6±16.4	0.07
Serum Creatinine (mg/dl)	0.9±0.2	0.86±0.2	0.85±0.18	0.41
Basal Insulin dose (U/die)	23.5±10.1	21.5±12.3	19.1±9.0	0.21
Bolus Insulin dose (U/die)	37.1±10.1	18.9±9.7	21.9±11.6	0.05

CSII, continuous subcutaneous insulin infusion; BMI, body mass index; HbA1c, glycated hemoglobin.

Data are expressed as mean ± SD.

**Table 3:** Overall CGM variables and Quality of Life questionnaire score.

	Group 1 (n=24)	Group 2 (n=49)	Group 3 (n=63)	P
***CGM variables***				
CGM use (%)	87.0±19.8	82.9±18.2	85.4±15.7	0.624
Mean glucose (mg/dl)	166.6±21.9	163.6±21.9	150±15.6	0.003
eHBA1c (%)	7.3±0.71	7.1±0.73	6.8±0.4	0.004
CV (%)	36.8±6.9	32.8±6.1	31.3±4.1	0.001
SD (mg/dl)	55.4±9.9	54.0±11.8	47.5±9.2	0.002
TBR 2 (<54 mg/dl)	1.35±2.3	0.45±0.6	0.18±0.5	0.000
TBR 1 (<70 mg/dl)	2.5±2.48	1.9±1.8	1.7±1.5	0.220
TIR (70-180 mg/dl)	59.1±13.6	62.3±17.20	70.6±12.9	0.002
TAR 1 (>180 mg/dl)	26.90±9.9	25.6±11.1	23.2±11.3	0.283
TAR 2 (>250 mg/dl)	10.7±8.1	9.3±11.3	4.5±4.7	0.003
***Questionnaire QOL score***				
Total	97.6±15.6	95.7±12.8	97.6±11.4	0.741
“Convenience” (CSII-QOL-C)	26.8±2.7	26.7±2.5	27.1±2.0	0.675
“Social restrictions” (CSII-QOL-SR)	39.2±6.8	39±6.6	39.5±6.3	0.945
“Psychological problem” (CSII-QOL-PB)	30.6±8.5	29.8±6.8	31.3±5.9	0.545

CGM, continuous glucose monitoring; CV, coefficient of variation; SD, standard deviation; TAR, time above range; TBR, time below range (%); TIR, time-in-range.

Data are expressed as mean ± SD.

## Discussion

The purpose of this cross-sectional, retrospective study was to evaluate benefits of different CSII systems, in terms of clinical outcome and quality of life, in real-life settings. A cohort of T1D patients on insulin pump treatment was divided into three groups, according to the type of CSII system used. All groups were comparable regarding sensor wear time, and all participants regularly used the automatic bolus insulin calculator feature, allowing a real comparison between the different categories. To our knowledge, there are no published QOL findings, with current available systems in real-life settings, and the examined population is quite large. The main limitations of this study are the retrospective nature, the lack of control group in MDI treatment, and the fact that participants had different timings of CSII initiation, however comparable between the three groups. Clinically significant differences were found in the subgroup of patients using hybrid close-loop and advanced hybrid close-loop systems. Participants of the HCL group showed a percentage of time spent in the euglycemic range of 11.5% higher than the SAP group, and 8.3% higher than the PLGS group, with 52.4% of subjects achieving the target range proposed by the international consensus on time in range (>70%) (13). These results agree with previous studies that showed similar differences of time in the euglycemic range, demonstrating an increase in TIR values between 5% and 10% with the HCL system (8, 14). The utility of the algorithm was again confirmed by a strong positive correlation, in the HCL group, between TIR values and time spent in auto-mode (r = 0.356 and p = 0.009). Reaching a higher percentage of time in the euglycemic range resulted in a consensual significant reduction of time spent both in hyperglycemia and hypoglycemia ranges. Exposure to the hyperglycemia range (>250 mg/dl) in the HCL group, was reduced by 6.2%, compared to the SAP group, and 3.1%, compared to the PLGS group, while the reduction was not significant between SAP and PLGS groups, confirming the effectiveness of basal insulin modulation in preventing values above the target range. The prevention of severe hypoglycemia (<54 mg/dl) was not different between HCL and PLGS groups, as expected, but both groups showed a significant reduction compared to the SAP group, -0.9% between SAP and PLGS and -1.17% between SAP and HCL systems. These data are similar to those obtained in the PROLOG and SMILE studies (15, 16) that reported a reduction of glycemia values <54 mg/dl between -0.1% and 3.3% with suspend before low technology, while Garg and colleagues reported a reduction of 0.5% of severe hypoglycemia passing from PLGS to HCL systems (8). The improvement in time spent in the euglycemic range and reduction of glycemia excursions resulted in lower values of glucose variability, expressed as coefficient of variation of CGM-measured glucose values, reduced by 14.9% in the HCL group compared to the SAP group. Thus, together with improvement in estimated HbA1c and mean glucose values, it permitted the HCL group to reach all targets of treatment proposed in the consensus of Advanced Technologies & Treatments for Diabetes (13). Regarding quality-of-life questionnaires, previous studies concluded that technological advancement, used to support people with T1D to manage their diabetes, is also associated with psychosocial benefits (17–20). Previous studies suggest a qualitative difference between using MDI and CSII which centers on experiencing metabolic improvements, feelings of ease, personal control, and confidence in habituating to more complex technology. The REPOSE trial, comparing CSII and MDI, focused on improvements in diabetes self‐management due to structured education and ongoing support, also indicating potentially stressful elements in introducing a new and complex technology into everyday life (21). Despite positive evidence regarding the impact of SAP use on QOL, compared to MDI (22, 23), little is known about how recent innovative pumps may influence QOL. Bergenstal et al. examined the impact of the LGS (low-glucose suspend) feature, compared to traditional SAP. LGS did contribute to a decrease in nocturnal hypoglycemia, but without any significant difference in QOL outcomes (24). Published data about QOL findings with HCL and AHCL pumps are still too limited and did not allow any solid conclusion. In our study, no significant differences were found in QOL among different types of insulin pumps; however, this was quite expected, as all subjects used CSII technology and there was a lack of control group in MDI treatment. Starting new pump therapy does take extra effort from both the diabetes team and the patient (21). Based on this, the negative correlation between perceived convenience in CSII use and higher CV values (r = -0.207, p = 0.043) underlines the relation between a better metabolic control and satisfaction for technology (20). In conclusion, our study demonstrates that HCL and AHCL systems provide better glycemic control, compared to standard sensor-augmented pumps but also to suspend before low technology, allowing a higher percentage of time in the euglycemic range, lower glucose variability, and lower hypoglycemic risk. These aspects, in particular the reduction of glucose variability, point to a promising trend in improving quality of life and higher acceptance of CSII systems, together with a reduction of acute and chronic complications related to diabetes disease.

## Data Availability Statement

The raw data supporting the conclusions of this article will be made available by the authors, without undue reservation.

## Ethics Statement

The studies involving human participants were reviewed and approved by Registro Sperimentazioni n 2020/ST/449, Comitato Etico Milano Area 1, ASST Fatebenefratelli Sacco, Milano Italy. The patients/participants provided their written informed consent to participate in this study.

## Author Contributions

PF, ML, AR, PM, and GZ designed the study. AG, LP, AB, LV, SA, and GZ collected the data. IC and ML performed the statistical analysis. LM, IP, VC, and ML wrote the draft manuscript. All authors contributed to the article and approved the submitted version.

## Conflict of Interest

The authors declare that the research was conducted in the absence of any commercial or financial relationships that could be construed as a potential conflict of interest.

## Publisher’s Note

All claims expressed in this article are solely those of the authors and do not necessarily represent those of their affiliated organizations, or those of the publisher, the editors and the reviewers. Any product that may be evaluated in this article, or claim that may be made by its manufacturer, is not guaranteed or endorsed by the publisher.
